# Effects of eHealth Interventions on Medication Adherence: A Systematic Review of the Literature

**DOI:** 10.2196/jmir.1738

**Published:** 2011-12-05

**Authors:** Annemiek J Linn, Marcia Vervloet, Liset van Dijk, Edith G Smit, Julia CM Van Weert

**Affiliations:** ^1^Amsterdam School of Communication ResearchUniversity of AmsterdamAmsterdamNetherlands; ^2^Netherlands Institute for Health Services ResearchUtrechtNetherlands

**Keywords:** Internet interventions, medication adherence, compliance, systematic review, tailoring, eHealth, effects, RCT

## Abstract

**Background:**

Since medication nonadherence is considered to be an important health risk, numerous interventions to improve adherence have been developed. During the past decade, the use of Internet-based interventions to improve medication adherence has increased rapidly. Internet interventions have the potential advantage of tailoring the interventions to the needs and situation of the patient.

**Objective:**

The main aim of this systematic review was to investigate which tailored Internet interventions are effective in improving medication adherence.

**Methods:**

We undertook comprehensive literature searches in PubMed, PsycINFO, EMBASE, CINAHL, and Communication Abstracts, following the guidelines of the Cochrane Collaboration. The methodological quality of the randomized controlled trials and clinical controlled trials and methods for measuring adherence were independently reviewed by two researchers.

**Results:**

A total of 13 studies met the inclusion criteria. All included Internet interventions clearly used moderately or highly sophisticated computer-tailored methods. Data synthesis revealed that there is evidence for the effectiveness of Internet interventions in improving medication adherence: 5 studies (3 high-quality studies and 2 low-quality studies) showed a significant effect on adherence; 6 other studies (4 high-quality studies and 2 low-quality studies) reported a moderate effect on adherence; and 2 studies (1 high-quality study and 1 low-quality study) showed no effect on patients’ adherence. However, most studies used self-reported measurements to assess adherence, which is generally perceived as a low-quality measurement. In addition, we did not find a clear relationship between the quality of the studies or the level of sophistication of message tailoring and the effectiveness of the intervention. This might be explained by the great difference in study designs and the way of measuring adherence, which makes results difficult to compare. There was also large variation in the measured interval between baseline and follow-up measurements.

**Conclusion:**

This review shows promising results on the effectiveness of Internet interventions to enhance patients’ adherence to prescribed long-term medications. Although there is evidence according to the data synthesis, the results must be interpreted with caution due to low-quality adherence measurements. Future studies using high-quality measurements to assess medication adherence are recommended to establish more robust evidence for the effectiveness of eHealth interventions on medication adherence.

## Introduction

Recent reports of the World Health Organization and the National Institute for Health and Clinical Excellence reveal that 30%–50% of patients with chronic illnesses do not adhere to prescribed medication [1]. Other studies also show that rates of nonadherence are very high and depend on the type of disease. The highest adherence rates are found for patients with human immunodeficiency virus infection, while diabetes patients have the lowest rate [2]. As such, medication nonadherence can be considered an important health care problem. This is especially true for patients with a chronic illness because medication adherence is a crucial factor in the effectiveness of a therapy [2]. Consequently, many patient-centered interventions are developed to improve adherence, and the impact of the Internet in the development of these interventions is increasing. It is therefore important to understand how these interventions work and to know whether they are effective in improving adherence. To our knowledge, no recent review has studied the effectiveness of patient-centered Internet interventions on patients’ medication adherence. Therefore, we conducted a systematic literature study in which we reviewed evidence from studies on Internet interventions that were developed to assist patients in their medication management. The purpose of our study was fourfold: first, to gain insight into the current stage of development of these interventions; second, to assess the included studies for their effectiveness on medication adherence; third, to investigate to what degree adherence is determined by the characteristics of the intervention; and fourth, to investigate whether there is a relationship between the characteristics of the study and the reported effectiveness of the interventions.

Different terms are used in the literature to describe the concept of adherence—for example, compliance, adherence, and persistence. They have all been used to indicate that the patient is using the medication following the prescribed regimen. These terms differ in exact meaning. In this paper, we use the term adherence. *Adherence* is defined as the extent to which the patient’s behavior matches the agreed recommendations of the prescriber [3]. According to this definition, nonadherence is a wide concept that varies from missing an occasional dose to never taking the prescribed medications [3]. Patients have different reasons for being nonadherent. These different reasons have something in common: the patient does not execute the treatment plan and does not persist. Execution is a continuous process where the actual dosing history corresponds to the ideal doses [4,5].

To improve adherence and develop target interventions, it is important to address the specific reasons why a patient is not able or willing to execute the treatment plan. From this perspective, interventions should be personalized or tailored to address individual needs and beliefs. The definition of tailoring describes the features that make tailored health messages different from other approaches: “It is assessment-based and as a result the message can be individual-focused*”* [6]. In other words, tailoring is based on gathering and assessing personal data related to health outcomes or several determinants in order to determine the most effective strategy to meet that person’s needs [6]. With these characteristics, a tailored message is able to provide personal feedback, commands greater attention, is processed more deeply, and is perceived as more likable by patients than a general message [7,8]. Because of these possibilities, tailored health messages are also more likely than generic information to be read, remembered, and viewed as personally relevant [6,9].

Computer technologies can be used to tailor health messages to the personal situation of the patient and might therefore contribute significantly to the development of tailored message strategies. The Internet is potentially a powerful medium for delivering those tailored messages. The management of a chronic disease should be personalized to an individual because the person is ultimately responsible for the success of the intervention [10]. The technology provides an opportunity to tailor the information in several formats and modalities, which enhances the user’s experience of the material and will result in a better understanding [7,8]. Moreover, Internet interventions have the advantage that they can provide interactive and responsive programs [10]. These interventions can provide effective data and information provision and retrieval. The advantages of tailored message strategies can contribute to the incorporation of interactive and continued self-monitoring, feedback, and information exchange, which play an increasingly important role in changing patients’ behavior.

## Methods

For this review, we used the guidelines of the Cochrane Collaboration to assess the studies on their internal validity and to summarize the existing evidence about Internet interventions to improve medication adherence in patients. The Cochrane Collaboration method is described in more detail in the Cochrane Handbook for Systematic Reviews of Interventions [11].

### Inclusion Criteria

We included a study when the following inclusion criteria were met: (1) the study described a patient-centered Internet intervention, (2) the study described an intervention for patients who use prescribed medication for a chronic condition, (3) at least one of the outcome measures was adherence, (4) the study was quantitative, and (5) the study was published in either the English or Dutch language.

### Search Methods

We conducted a systematic literature search to identify articles containing information about the effect of Internet interventions on medication adherence. Comprehensive literature searches were undertaken in the databases PubMed, PsycINFO, EMBASE, CINAHL, and Communication Abstracts.

The search strategies used the following keywords: (medication therapy management OR medication adherence OR patient compliance OR self-care) AND (internet) AND (intervention study OR randomized controlled trial OR clinical controlled trial).

We then continued with the snowball method by looking for references in publications, especially those of the included studies and reviews on interventions to promote adherence. The search was conducted in June 2010. Since Internet intervention is a relatively new topic, no time limits were applied.

Application of the search strategy to the specified databases resulted in a total of 620 hits (Table 1). In total, we selected 13 studies from these results.

**Table 1:** Results of database searches.

Source	Hits per strategy	Unique studies	Relevant studies
PubMed	388	388	11
Communication Abstracts	0	0	0
PsycINFO	47	40	0
EMBASE	169	82	0
Snowball method	3	0	1
CINAHL	13	10	1
Total	620	520	13

**Figure 1:**
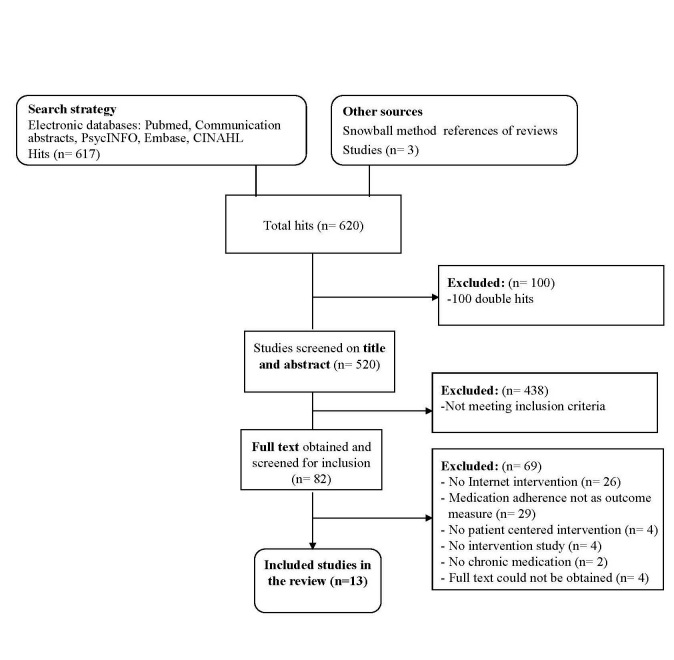
Flow diagram of study search and selection.

Reference Manager version 11.0 (Thomson Reuters, New York, NY, USA) was used to manage the citations. Duplications were logged, leaving 520 unique results (see Figure 1). On the basis of title and abstract, two researchers (pairs of AL, MV, LvD, JvW) independently selected studies for inclusion. If the study seemed to meet the inclusion criteria or if there were doubts about the inclusion, the full text of the article was obtained. Based on the full articles two reviewers independently reviewed whether these studies fit all the inclusion criteria. Disagreements were solved by discussions between the two researchers. For a more detailed description of the excluded studies see Multimedia Appendix 1.

### Assessment of Methodological Quality

The methodological quality of included randomized controlled trials (RCTs) and clinical controlled trials (CCTs) was independently reviewed by two researchers (AL and JvW) using the list from the Cochrane Collaboration Back Review Group [12]. The list consists of 11 criteria for internal validity, namely:

- 3 criteria regarding selection bias: whether (a) randomization was adequate, (b) treatment allocation was concealed, and (c) groups were similar at baseline regarding the most important indicators,
- 4 criteria for performance bias: whether (d) patients were blinded to the intervention, (e) care provider was blinded to the intervention, (g) co-interventions were avoided, and (h) compliance with the intervention was acceptable,
- 2 criteria regarding attrition bias: whether (i) the dropout rate after baseline was acceptable, and (k) the analysis included an intention-to-treat analysis, and
- 2 criteria for detection bias: whether (f) the outcome assessor was blinded to the intervention, and (j) outcome assessments in all groups were similar.

For each included study, all criteria were scored as “yes,” “no,” or “unclear.” All unclear scores were later rated as “no.” Studies were rated as high quality (HQ) when at least 6 of the 11 criteria for internal validity were met. Otherwise, studies were considered of low quality (LQ). Disagreements were discussed until consensus was reached. If disagreement or indistinctness persisted a third reviewer (LvD) was consulted.

In addition, two researchers (AL and LvD) independently assessed the quality of the methods for measuring adherence to a medical regimen. A standard method to assess adherence does not exist and every method has its limits [13,14]. In clinical trials, adherence can be measured based on, for example, interviews, diary, questionnaire-based self-reporting, prescription refills, pill counts, or electronic monitoring [14,15]. We categorized the measurements in high- and low-quality adherence assessments based on previous findings concerning the objectivity of these measurements [13-15]. In this review electronic monitoring and physiological/biomedical measures are defined as high-quality adherence assessment. These measurements are considered the most objective standard [15]. Previous research has shown that data from pill counts and electronic monitoring are strongly correlated [16]. Yet others consider pill counts not to be accurate [17,18]. In addition, meta-analyses have shown that self-reported adherence is also strongly correlated with electronic monitoring [19,20]. Like pill counts, the accuracy of self-reported measurements is debatable. Some argue that self-reports may be an accurate measurement for measuring adherence [21,22], while others state that the use of self-reported measurements is not an accurate method [14,15,23,24]. Taking all arguments into account, we considered self-measurements, such as questionnaires, pill counts, prescription refills, interviews, and diaries, to be most subjective for measuring adherence [13]. We therefore considered these measurements low-quality adherence assessment. However, if two or more different low-quality adherence measurements were used in the same study, such as a combination of questionnaires and prescription refills, the method was considered high-quality adherence assessment.

### Data Extraction

One researcher (AL) documented the following characteristics of the included studies: (1) method (type of study), (2) participants (total number of participants, sex per group, mean age per group, type of disease), (3) intervention (name of experimental intervention, name of control condition, period, number of times/minutes per week), (4) outcome measures (type of outcome measures, time of measurement), (5) results (short description), and (6) author’s conclusion.

### Data Synthesis

Due to diversity in the features of the interventions and the methods used to measure adherence, it was not possible to pool the data. Therefore, we conducted a best-evidence synthesis (see Textbox 1) based on [12] and adapted by a Dutch study [25].

The best-evidence synthesis was conducted by attributing various levels of evidence to the effectiveness of the interventions. The synthesis takes into account the design, methodological quality, and outcomes of the studies. Textbox 1 shows that at least 1 HQ RCT or 2 HQ CCTs were needed to establish robust evidence for the effectiveness of an intervention.

**Principles of Best-Evidence Synthesis.**

Evidence:

Provided by consistent, statistically significant findings in outcome measures in at least 2 high-quality randomized controlled trials (RCTs).

Moderate evidence:

Provided by consistent, statistically significant findings in outcome measures in at least 1 high-quality RCT and at least 1 low-quality RCT or high-quality CCT.

Limited evidence:

Provided by statistically significant findings in outcome measures in at least 1 high-quality RCT

Or

Provided by consistent, statistically significant findings in outcome measures in at least 2 high-quality CCTs (in the absence of high-quality RCTs).

Indicative findings:

Provided by statistically significant findings in outcome measures in at least 1 high-quality CCT or low-quality RCT (in the absence of high-quality RCTs).

No/insufficient evidence:

If the number of studies that have significant findings is <50% of the total number of studies found within the same category of methodological quality and study design

Or

In case the results of eligible studies do not meet the criteria for one of the above stated levels of evidence

Or

In case of conflicting (statistically significantly positive and statistically significantly negative) results between RCTs and CCTs

Or

In case of no eligible studies.

### Sensitivity Analysis

We conducted a sensitivity analysis to indentify how sensitive the results of the best-evidence syntheses were to changes in the way the study quality was assessed. For the sensitivity analysis, the best-evidence synthesis was repeated in two different ways, using the following principles: (1) LQ studies were excluded, (2) studies were rated as HQ if they met at least 4 of the 11 criteria of internal validity instead of 6.

We then compared the results of the sensitivity analysis with the results of the best-evidence synthesis and described the sensitivity of the results [25,26].

### Effectiveness

Study effectiveness was categorized as significant effect on adherence, moderate effect on adherence, and no effect on adherence. We defined a study effect as moderate if the authors reported a positive effect of the intervention on adherence but there were limitations, such as the following: improvement of adherence was found only in a subgroup of the intervention group; adherence was measured indirectly (eg, the study drew conclusions about the use of beta-agonist indicating that adherence was improved); or the significance of the results to medication adherence was not tested, but the authors used convincing arguments to explain the effectiveness of the intervention (see results section for explanation per study).

### Intervention: Tailoring Level of Sophistication of the Website

**Figure 2:**
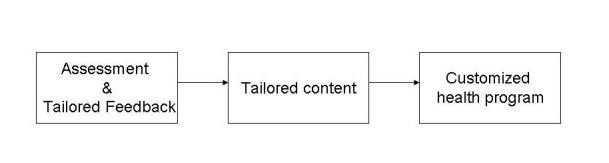
Continuum level of sophistication of tailored intervention.

Tailored Internet interventions differ in how they deliver their message [7]. The difference is based on the sophistication of the way the message is tailored. We categorized the interventions in being low, moderate, or high in sophistication. Some interventions involve a form of online assessments (low sophistication), and others use online assessments, tailored feedback, and content matching (moderate sophistication). The third group of interventions provides instant feedback and a complex tailored health program with several tools and activities that would enable patients to achieve their health goals (high sophistication) (see Figure 2) [7].

## Results

The main characteristics of the included studies are presented in Table 2 [27-39] and further described below (for a more detailed description of the included studies see Multimedia Appendix 2).

**Table 2:** Characteristics of included studies.

Study; method	Intervention^a^	Participants; sex; mean age	Adherence measurement; timing of measuring adherence	Main conclusion
Artinian [27]; RCT^b^	Web-based monitoring system; tailored content; nature of expert/therapist contact	N = 18 (17 males; mean age 68 years); intervention group n = 9, control group n = 9	Pill counts; baseline and 3 months	Medication compliance rate was 94% for the monitor group as measured by the monitor system
Jan [28]; RCT^b^	Blue Angel for Asthma Kids variability; tailored content; nature of expert/therapist contact	N = 164, intervention group n = 88 (35 males; mean age 10.9 years); control group n = 76 (28 males; mean age 9.9 years)	Self-reported at baseline and 12 weeks	The Blue Angel for Asthma Kids has the potential for improving asthma outcome compared with conventional treatment over a period of 12 weeks
Chan [29]; RCT^b^	Customized educational and monitoring Web site; tailored content; nature of expert/therapist contact	N = 120; intervention group n = 60 (37 males; mean age 10.2 years); control group n = 60 (38 males; mean age 9.0 years)	Computerized prescription refill record at baseline, 26 weeks, and 52 weeks	No difference in adherence between groups
Chan [30]; RCT^b^	Customized educational and monitoring Web site; tailored content; nature of expert/therapist contact	N = 10; intervention group n = 5 (1 male; mean age 6.6 years); control group n = 5 (4 males; mean age 8.7 years)	Self-reported asthma diary and computerized prescription refill record at 90 days and 180 days	After the intervention, the use of beta-agonist decreased, which is an indication of better adherence
Joseph [31]; RCT^b^	Web-based asthma management program; tailored content; user control	N = 314 (36.6% male; mean age 15.3 years); intervention group n = 162; control group n = 52	Self-reported at baseline and 12 months	Positive changes in controller medication adherence were seen
Ross [32]; RCT^b^	SPPARO (System Providing Access to Records Online); tailored content; nature of expert/therapist contact	N = 104; intervention group n = 54 (80% male; mean age 57 years); control group n = 50 (74% male; mean age 55 years)	Self-reported at baseline, 6 months, and 12 months	Providing patients access to an online medical record improved adherence
Cherry [33]; prospective design	Telemedicine diabetes disease management program; tailored content; nature of expert/therapist contact	Intervention group n = 169 (39 males; mean age 53 years); historical group (usual care)	Self-reported	Outcomes offer encouraging evidence that telemedicine technology coupled with daily remote monitoring may improve appropriate use of health care services
Guendelman [34]; RCT^b^	Health Buddy, an interactive device connected to a home telephone; tailored content; nature of expert/therapist contact	N = 134; intervention group (40 males; mean age 12.2 years); control group (37 males; mean age 12.0 years)	Self-reported at baselines, 6 weeks, and 12 weeks	Patients were more likely to take their asthma medication without additional reminders
DeVito Dabbs [35]; RCT^b^	Pocket Personal Assistant for Tracking Health (PATH); tailored content; nature of expert/therapist contact	N = 30; intervention group n = 15 (60% male; mean age 55 years); control group n = 15 (60% male; mean age 57 years)	Self-reported at baseline and 2 months	Patients who received the PATH were more likely to show high adherence to the medical regimen
Van der Meer [36]; RCT^b^	Internet-based self-management program; customized health program; user control	N = 200; intervention group n = 101 (29% male; mean age 36 years); control group n = 99 (29% male; mean age 37 years)	Self-reported at baseline, 3 months, and 6 months	After 3 months asthma control improved
Van der Meer [37]; RCT^b^	Internet-based self-management program; customized health program; user control	N = 200; intervention group n = 111 (28 males; mean age 36 years); control group n = 89 (28 males; mean age 36.6 years)	Self-reported at baseline, 3 months, and 1 year	Weekly self-monitoring leads to improved asthma control in patients with partly and uncontrolled asthma at baseline and tailors asthma medication to individual patients’ needs
Dilorio [38]; Survey	WebEase; customized health program; user control	N = 35 (40% male; mean age 37.5 years)	Self-reported at baseline and 6 weeks	Participants showed some improvement in adherence following the program
Dew [39]; prospective design	Website including skills workshops, discussion group, ask an expert, question and answer, health tips, recourses, and references; customized health program; nature of expert/therapist contact	N = 64; intervention group n = 24 (18 males; mean age 45.8 years); control group n = 40 (30 males; mean age 57.5 years)	Self-reported at baseline and 4 months	The intervention appeared to be weakly associated with medical compliance change

^a^ Sophistication of tailoring classification based on Figure 2.

^b^ Randomized controlled trial.

### Methodological Quality: Assessment of Internal Validity

For this review 10 RCTs were included, and 9 of them were assessed on their internal validity (Table 3 [27-39]). We included 1 RCT with no data on our primary outcome variable (ie, medication adherence) for the control group [27]. This means that for this tenth study, we could not assess validity criteria. Moreover, we reviewed 2 prospective cohort designs and 1 survey. A total of 7 RCTs met 6 or more of the 11 validity criteria and therefore qualified as HQ studies.

**Table 3:** Results of methodological quality.

Study		Validity criteria^a^ met	Study quality^b^	Quality measurement adherence
**Randomized clinical trials**				
	Artinian [27]	Not applicable^c^	Low	Low
	Jan [28]	a, b, c, d, i, j	High	Low
	Chan [29]	a, b, f, i, j	Low	Low
	Chan [30]	a, b, c, f, h^d^, i, j	High	High
	Joseph [31]	a, b, c, d, e, h, i, j	High	Low
	Ross [32]	a, b, c, d, e, h^e^, i, j, k	High	Low
	Guendelman [34]	a, b, d, i, j	Low	Low
	DeVito Dabbs [35]	a, b, c, e, i, j	High	Low
	Van der Meer [36]	a, b, c, i, j, k	High	Low
	Van der Meer [37]	a, c, h^f^, i, j, k	High	Low
**Prospective design/clinical trial or cohort design**				
	Cherry [33]		Low	Low
	Dew [39]		Low	Low
**Survey**				
	Dilorio [38]		Low	Low

^a^ a: randomization adequate; b: treatment allocation concealed; c: groups similar at baseline regarding most important indicators; d: patients blinded to intervention; e: care provider blinded to intervention; f: outcome assessor blinded to intervention; g: co-interventions avoided; h: compliance with intervention acceptable; i: dropout rate after baseline acceptable; j: outcome assessed similarly in all groups; k: intention-to-treat analysis included.

^b^ That is, 6 of 11 validity criteria were met.

^c^ No data on medication adherence for the control group and therefore judged as low quality.

^d^ Compliance was acceptable in the first interval (<90 days).

^e^ Compliance was acceptable in the first interval (6 months).

^f^ Compliance was acceptable in the first interval (3 months).

### Intervention: Tailoring Level of Sophistication of the Website

All Internet interventions reported computer-tailoring methods. Interventions were categorized as having low sophistication (online assessments), moderate sophistication (online assessments, tailored feedback, and content matching), and high sophistication (a more complex tailored health program) (see Figure 2) [7].

#### Online Assessment and Feedback

Online assessment and feedback are used in interventions with single-incident computer-assisted risk or health assessments. For example, feedback is emailed to the patients or provided online [7]. In addition, these interventions are brief and usually done once at the beginning of the intervention. None of the reviewed studies used online assessment and feedback.

#### Tailored Content

With tailored content a program provides (1) tailored text messages composed in a unique way according to how patients respond to certain questions, or (2) restricted access to content sections per patient [7]. Tailored content was used by 9 of the studies we reviewed; 1 study [28], Blue Angel for Asthma Kids, conducted an Internet-based interactive asthma educational and monitoring program in which patients were able to complete an electronic diary, record symptoms and need for rescue medication, and upload their videos when they were using their inhaler. Based on these outcomes, the program comprised both an action plan with a warning system and a written treatment plan. A similar customized educational and monitoring Website for patients with asthma was conducted in 2 studies [29,30], and 1 study [31] tested the asthma management program Puff City. The program used tailoring to alter behavior through individualized health messages based on the patients’ beliefs, attitudes, and personal barriers to change or maintain the behavior. Another form of a tailored website, System Providing Access to Records Online (SPPARO), was examined in 1 study [32]. This website provided the medical record, an educational guide, and a message system for patients. Moreover, patients could contact the health provider by email. The telemedicine diabetes disease management program Health Buddy was tested in 2 studies [33,34]. However, the modality used in the diabetes program was different from the previously mentioned monitoring programs. Patients answered personalized questions that enabled them to monitor their disease symptoms, medication adherence, and disease knowledge by pressing buttons for response. The 2 studies using the Health Buddy differed in the intensity of the feedback. One study [27] tested a medication compliance device. Data and answers to questions were recorded by the device and uploaded daily to a central server. Based on these answers health providers were able to monitor the patients, provide advice, and update the treatment regimens in the Med-eMonitor devices. One study [35] also tested a handheld device, Pocket Personal Assistant for Tracking Health (PATH), developed for patients after lung transplantation to record health data, review data trends, and report their condition changes to the transplant team. The device included decision-support programs to promote self-care behaviors.

#### Customized Health Programs

Interventions that provide not only tailored content but also individualized instructions for meeting certain health goals, self-management goals, or goal-setting activities are so-called customized health programs [7], used by 4 of the included studies. Of these, 2 studies [36,37] tested the effects of an Internet-based self-management program for asthma patients. This website allowed monitoring through the website, text messages, use of an Internet-based treatment plan, online education, and the possibility to communicate with the health provider. The intervention WebEase [38] consisted of three modules that were designed to assess an individual’s status related to self-management practices and create a plan for change or to maintain the behavior. The modules in WebEase required the patient to answer questions related to these topics. Feedback was provided based on these responses. Patients entered data into MyLog, which is a screen for recording data about medication-taking behavior, stress, etc. In addition, the intervention included a knowledge component and a discussion board. This means that each patient was directed to another path [38]. Another study [39] tested a customized health program where patients chose which components of the website they wanted to use. The website included a home page, posttransplant skills workshops, discussion groups, “ask an expert,” question-and-answer possibility, healthy-living tips, resources, and reference library. The way the patients used the website was based more on voluntary participation than in the study that used MyLog [38].

Table 4 and Table 5 show for each study which method for delivering the tailored message was used (see column 2).

**Table 4:** Effectiveness of short-term interventions (<6 months).

Study	Study quality	Sophistication of tailoring	Quality measurement adherence	Short-term effectiveness (<6 months)^a^
DeVito Dabbs [35]	High	Moderate	Low	++
Jan [28]	High	Moderate	Low	++
Dew [39]	Low	High	Low	–
Dilorio [38]	Low	High	Low	++
Artinian [27]	Low	Moderate	Low	+
Guendelman [34]	Low	Moderate	Low	++

^a^ ++ = significant effect on medication adherence; + = moderate effect on medication adherence; – = no effect on medication adherence.

**Table 5:** Effectiveness of long-term interventions (>6 months).

Study	Study quality	Sophistication of tailoring	Quality measurement adherence	Long-term effectiveness (>6 months)^a^
Van der Meer [36]	High	High	Low	+
Van der Meer [37]	High	High	Low	+
Chan [30]	High	Moderate	High	+
Joseph [31]	High	Moderate	Low	++
Ross [32]	High	Moderate	Low	+
Chan [29]	Low	Moderate	Low	–
Cherry [33]	Low	Moderate	Low	+

^a^ ++ = significant effect on medication adherence; + = moderate effect on medication adherence; – = no effect on medication adherence.

#### Role of Health Providers

Interventions also differ in the type and extent of health provider involvement. User control allows individuals to take a major role in managing their own care, whereas in expert control an expert or therapist takes a more directive role [7].

Only 4 of the interventions were based on user control and 9 interventions used contact with the health provider. A Web-based asthma management program was developed in 1 study [31]. The program used tailoring to alter behavior through individualized health messages based on the user’s beliefs, attitudes, and personal barriers to change. The health provider did not interfere. Three studies were also user based with treatment algorithms to give feedback [36-38].

In contrast, in the Blue Angel for Asthma Kids, a customized educational and monitoring website site providing secure email contact between patients and their therapist, the therapist had a more directive role [28]. Like the Blue Angel for Asthma Kids, SPPARO included a messaging system that made it possible to exchange secure messages with the health provider [32]. The intervention developed in 2 studies [29,30] consisted of a case manager who reviewed the data, sent emails about the peak flow, inhaler technique, and symptoms, and forwarded them the website. Patients (the virtual group as well as the office-based group) had access to their case manager 24 hours a day, 7 days a week. One study [39] conducted a website including skills workshops, discussion group, ask an expert, question and answer, health tips, recourses, and references. In this case, the role of health providers was to provide the possibility for a patient to ask an expert.

Several interventions used a special device to exchange information between patients and health care providers. The Med-eMonitor recorded data and answers to questions that could be uploaded by the health provider. Based on these outcomes, the health provider provided advice and updated the treatment regimens [27]. The PATH [35] recorded data and provided a tailored decision-support program and email contact with the health provider. The Health Buddy device in 2 studies [33,34] is like the Med-eMonitor and PATH based on the feedback and participation of the health provider, but there is a difference in the intensity of participation of the health provider. In 1 study [33] the health provider contacted the patient only when necessary, while in the other 2 studies [34,35] the patient received feedback instantly after sending a question.

Our analysis to examine the extent to which medication adherence is determined by different tailoring levels revealed no clear relationship between the intervention’s level of sophisticated tailoring and the extent to which the intervention was effective (Table 4).

### Summary of Effects on Adherence

We found 5 studies with a significant effect on adherence (3 HQ studies and 2 LQ studies) [28,31,34,35,38]. The first study [28] concluded that the intervention had a positive and significant effect on use of the inhaled corticosteroid and that this effect significantly differed from the baseline. In addition, the second study [31] found positive changes in controller medication adherence. The third study [38] tested the WebEase intervention and found a significant effect on adherence. The fourth study [34] found that patients were more likely to take their asthma medication when they used the Health Buddy. In the fifth study [35], patients who received the PATH intervention were more likely to adhere to their medical regimen.

A moderate effect on adherence was reported in 6 studies (4 HQ studies and 2 LQ studies). The SPPARO proved to be feasible and improved general adherence. Adherence to medication showed a similar trend but these results did not reach significance [32]. The second study [30] concluded that, after the intervention, the use of beta-agonist decreased, which is an indication of better adherence. The third study [33] reported that medication compliance improved from 65% at pretest to 94% at posttest, but the difference was not statistically tested. The medication compliance rate in the fourth study [27] was 94% for the monitor group as measured by the monitor system. However, because there was no pretest and the data of the control group were not available, it is unknown whether the results were significant as compared with the pretest or the control group. The intervention of the last 2 studies [36,37] improved adherence in patients with partly and uncontrolled asthma at baseline. The authors concluded that the intervention was most effective in improving adherence for patients with partly or uncontrolled asthma at baseline.

No significant results on patients’ adherence were found in 2 studies (1 HQ and 1 LQ) [29,39].

### Assessment of Adherence Measurements

Regarding the measurement of adherence, the 13 studies we reviewed showed a large variability of methods: 12 studies used a low-quality measurement to assess adherence and 1 used a combination of these methods (ie, a high-quality measurement to assess adherence) (Table 3).

#### Low Quality of Adherence Measurement

In 10 studies, self-reported scales were used to obtain the adherence rate. Although 5 studies [28,31,34,36,37] used self-reports to measure adherence, they did not describe what kind of instrument they used. One study [39] used self-reported data by asking questions regarding adherence during the initial interview. Reports of therapist and patients were compared. In addition, 1 study [33] used a self-developed medication adherence survey on the Health Buddy appliance. The other 3 studies chose existing, valid, self-reported adherence scales. One study [32] used a combination of the Morisky scale and the General Adherence Scale from the Medical Outcomes Study, and 1 study [35] used the Health Habits Assessment, a self-reported scale to measure adherence. One study [38] used the self-report USCF Adherence Questionnaire and the Antiretroviral General Adherence Scale. Finally, 2 studies used measurements such as counting pills [27], and 1 study [29] used a computerized prescription refill record (after evaluation of the pilot study in which completing the diary turned out to be time consuming and inconvenient; see [30]).

#### High Quality of Adherence Measurement

One study used a combination of methods. This study [30] used a diary in combination with a computerized prescription refill record.

Table 3 shows the results of the assessment of the internal validity and the quality of adherence measurement.

### Relation Between Quality of Adherence Measurement and Effectiveness

Our investigation of the relationship between the quality of the adherence measurement and the effectiveness of the interventions revealed no clear relationship (there was only 1 study using a high-quality method to assess adherence), although self-reported adherence measurements seemed to result more often in significant effects than did pill counts and pharmacist adherence measurements (Table 4 and Table 5). Of the 10 studies using self-reports (low-quality adherence measurement), 5 reported a significant effect of the intervention on adherence [28,31,34,35,38], 4 a moderate effect [32,33,36,37], and 1 no effect [39]. From the 2 studies in which pharmacist data or pill counting was used, 1 reported a moderate effect [27] and 1 no effect [29]. The 1 study that used a combination of methods to measure adherence [30] found a moderate effect on adherence.

### Relation Between Interval of Adherence Measurement and Effectiveness

There was no clear relationship between the timing of the adherence measurements and the effectiveness of the intervention. The intervals between baseline and follow-up measurements differed between projects. Short-term adherence (ie, within 6 months) was measured in 6 studies. The first study [38] showed that WebEase improved adherence 6 weeks after baseline. Patients who used PATH were more likely to show higher adherence than the control group after an interval of 8 weeks after baseline [35]. The third study [28] found a significant effect on adherence after 12 weeks, and the fourth study [34] reported an improvement in adherence after 12 weeks. The fifth study [27] found an adherence rate of 94% in the experimental group after 12 weeks. Because of the lack of adherence data for the control group and the lack of a pretest, the effects on adherence could not be established. The sixth study [39] examined the proportion of nonadherent patients in both an intervention and a control group after 16 weeks. That study’s authors found uniformly small and nonsignificant differences between the control and intervention groups. However, they found an important difference within the intervention group. Subgroup differences appeared when the intensity of using parts of the intervention was related to the effectiveness. For example, patients who used the Managing Medical Regimen Workshop more often or intensely appeared to be more adherent than those using the intervention less often or intensely [39].

Long-term adherence was measured in 7 studies—that is, adherence with an interval of 6 months or longer, mostly of 1 year or more. Two studies [30,36] reported a moderate effect in their pilot on adherence after 6 months. In 2 studies [31,32], they found a moderate [32] and significant [31] improvement in adherence after 1 year. Two studies [33,37] found a moderate effect on adherence after 1 year. This means that all of the included studies using an interval of 6 months or longer showed an effect (significant or moderate) on long-term adherence. One study [29] did not find an effect on adherence after 1 year.

Table 4 and Table 5 give an overview of the methodological quality of the studies, the level of sophistication of each intervention, the quality of measurement of adherence, and an overview of the short-term and long-term effects.

### Data Synthesis

Using the principles of the best-evidence synthesis (see Textbox 1), taking into account the design, methodological quality, and outcomes of the studies, the following conclusions can be drawn. In total, 7 studies were considered HQ. We found 3 HQ studies [28,31,35] and 2 LQ studies [34,38] that had a significant effect on enhancing medication adherence and that met 6 of 11 criteria. This means that there is evidence that tailored Internet interventions are successful in improving medication adherence.

### Sensitivity Analysis

The sensitivity analysis showed the same results as the best-evidence synthesis. The results remained the same when the analysis was repeated with the 6 LQ studies excluded (ie, taking only the 7 HQ studies into account). Moreover, when studies were rated to be HQ if 4 instead of 6 criteria of interval validity were met, results stayed the same.

## Discussion

### Principal Results

First, our objective was to gain insight into the current state of the use of Internet interventions to improve medication adherence. Results of this review indicate that this is still a new field. This is visible in the differences in interventions with respect to crucial aspects such as the level of sophisticated tailoring and the role of health care providers. Despite the differences, it is remarkable that none of the interventions used a low level of tailoring and the majority (9 of 13) provided the opportunity to contact a health provider.

Second, the studies were assessed on their effectiveness on medication adherence. There is evidence that Internet interventions can improve medication adherence. This evidence comes from 3 HQ studies and 2 LQ studies, finding significant results on medication adherence.

Third, we wanted to investigate to what degree adherence is determined by the characteristics of the intervention. All interventions discussed in this review used tailored methods and used a moderately or highly sophisticated tailored intervention. These types of health programs, especially customized health programs, are more complex, generally long-term, allowing the patients to access the programs several times [7], and are considered appropriate for difficult-to-influence behaviors. We did not find a clear relationship between how sophisticated the tailoring of the intervention was and the extent to which the intervention appeared to be effective, possibly due to the various methods that were used.

Last, we wanted to investigate whether there is a relationship between the characteristics of a study and the reported effectiveness of the interventions. We found that there was variation not only in the level of tailoring, but also in the measurement of adherence, the timing of measuring adherence, and the intensity of the intervention. The included studies used self-reporting measurements (ie, interviews, diary, self-reporting via questionnaires) or pill counts, or prescription refills, or a combination. No study used electronic monitoring, which is perceived as a high-quality method for assessing adherence [15]. Of the 13 studies we reviewed, 7 measured long-term adherence, using an interval of 6 months or longer. There is no clear evidence that the duration of the intervention is related to the effectiveness of the intervention. Nevertheless, of the 7 studies measuring long-term adherence, 1 HQ study showed positive effects and 4 HQ studies and 1 LQ study showed moderate effects on adherence. This indicates that long-term interventions are promising. However, more research in this field is needed.

There is evidence that Internet interventions can be effective in improving medication adherence. The evidence comes from 3 HQ studies. However, the results should be interpreted with caution. Self-reported scales were used in 10 studies, which is considered a low-quality adherence measurement: 5 reported a significant effect of the intervention on adherence, 4 a moderate effect, and 1 no effect. Self-measurements can contribute to overestimating of the effects of interventions [40]. This could be explained by the possibility that patients may forget that they missed a dose. Biases that appear most prominent in estimating adherence by the patient from structured questionnaires are social desirability and social approval [5,13]. In other words, studies relying on self-reporting may have a tendency to err on the optimistic side when it comes to adherence, certainly compared with more objective pill-counting studies. However, it must be noted that anonymous self-report questionnaires are found to be significantly correlated with electronic monitoring [41] and virologic response [40], considered more objective methods. Research also shows that using specific strategies, such as ensuring patients that their responses will be kept confidential [40] or stratifying patients according to their socially desirable response [42], improves the prediction of adherence by self-reports. This indicates that self-reports are not useless, but future research should examine more strategies to reinforce accurate reporting by patients [40]. On the other hand, we included RCTs and, consequently, self-reported adherence can be expected to be overestimated in both treatment arms. Thus, the intervention effect (ie, difference between intervention and control group) was not necessarily overestimated. Additionally, a distinction can be made between valid self-report measurements and measurements that are not. If self-reported measurements are used, for instance because this is a cost-effective method, using validated measurements is recommended.

Electronic monitoring or observation is considered to be more accurate. It electronically records the time and date of the actual dosing events. Because every single method has its limitations, the best approach is to use multiple assessment techniques concurrently, as a way to improve the accuracy of adherence assessment [14]. One study in our review [30] used self-reported diary and prescription refills to measure adherence and made a distinction in interpreting the results. They used a self-reported diary to assess how the patients used their inhaler, based on the idea that when patients are not using their inhaler according to the health providers’ advice, they can be considered nonadherent. In addition, they used prescription refills to measure how many refills the patient was obtaining. In line with this method of measuring adherence, the optimal approach could be suggested to be a combination of self-reports and more objective measurements [43]. In addition, every single measurement needs a different interpretive approach because it has different relationships to clinical outcomes [13].

Of the studies we reviewed, 6 measured short-term adherence that varied from 6 weeks to 4 months: 4 of them were found highly effective and 1 moderately effective regarding adherence. The question is whether adherence improved in the long term, because the period was too short to measure persistence. According to an international expert forum on adherence [13], it is not easy to identify adherent and nonadherent patients beforehand. There is a large body of evidence dominated by reports identifying factors that are predictive or associated with nonadherence. Adherence could be seen as a dynamic behavior that is determined or influenced by unrelated factors that fluctuate and change over time [44]. As an adherent patient can become nonadherent over time, the importance of time (ie, persistence) has been emphasized [5]. The quality of execution of the treatment plan can influence persistence. Factors such as perceptions of treatment outcomes, beneficial effects, and adverse effects can influence the quality of execution over time. Therefore, conducting interventions that address long-term adherence and overcome reasons why a patient is not able or not willing to adhere are recommended.

### Methodological Limitations

The search method was top-down in that we relied on existing databases and search terms. This approach has the possibility of missing important articles due to miscoding of search terms. A bottom-up strategy is more time consuming but has the advantage of being more comprehensive.

### Clinical Implications

Monitoring adherence to optimize effects and minimize nonadherence could be time consuming. Computers, however, are very good at collecting data concerning the monitoring of adherence. Internet interventions can be tailored, collect data, and monitor adherence. In addition, based on this systematic literature review, there is evidence that tailored Internet interventions can be an effective method to improve medication adherence. This means that Web-based interventions can be effective at increasing medication adherence among chronically ill patients. Health providers, who want to enhance patients’ adherence, are encouraged to use tailored websites or reminder systems. They could use these interventions in addition to their everyday work.

### Implications for Research

Because we did not find a clear relationship between the effectiveness and the degree of tailoring, we recommend that future studies should be conducted with variation in the level of sophistication of tailoring to further test which characteristics of the tailored messages have the most positive effects on adherence. Moreover, website compliance is often not completely reported. While the frequency in which the patient used the website is often reported, studies do not describe how exactly patients used the website. Therefore, it is difficult to compare the results of different interventions, because the way patients use the website can have implications for the effectiveness of the website.

### Conclusion

With more than 40 million people using the Internet for a variety of purposes, health communication programs in the future are more likely to be delivered online [6]. These types of interventions especially have the potential to address difficult-to-change behaviors such as adherence. This review shows promising results on the effectiveness of tailored Internet interventions to enhance medication adherence of chronically ill patients. There is evidence that these interventions can enhance adherence. But it remains a relatively new field, and studies using more objective measurements to assess adherence are recommended.

## Multimedia Appendix 1

Detailed description of the excluded studies.

## Multimedia Appendix 2

Detailed description of the characteristics of the included studies.

## Abbreviations

CCT: clinical controlled trial
HQ: high quality (study)
LQ: low quality (study)
PATH: Pocket Personal Assistant for Tracking Health
RCT: randomized controlled trial
SPPARO: System Providing Access to Records Online
