# Supplementary material for: Effects of eHealth Interventions on Medication Adherence: A Systematic Review of the Literature
Source: J Med Internet Res. 2011 Dec 5;13(4):e103. doi: 10.2196/jmir.1738 (PMC3278089; doi:10.2196/jmir.1738)
Supplement: Supplementary file 1 [file jmir_v13i4e103_app1.pdf]

Multimedia Appendix 1

	Database	Authors	Title	Reason for exclusion
1.	PubMed	Bender BG, Apter A, Bogen DK, Dickinson P, Fisher L, Wamboldt FS, Westfall JM.	Test of an interactive voice response intervention to improve adherence to controller medications in adults with asthma.	No internet intervention
2.	PubMed	Watson AJ, Kvedar JC, Rahman B, Pelletier AC, Salber G, Grant RW.	Diabetes connected health: a pilot study of a patient- and provider-shared glucose monitoring web application.	Medication adherence not as outcome measure
3.	PubMed	Mulvaney SA, Rothman RL, Wallston KA, Lybarger C, Dietrich MS.	An internet-based program to improve self-management in adolescents with type 1 diabetes.	Medication adherence not as outcome measure
4.	PubMed	Svetkey LP, Pollak KI, Yancy WS Jr, Dolor RJ, Batch BC, Samsa G, Matchar DB, Lin PH.	Hypertension improvement project: randomized trial of quality improvement for physicians and lifestyle modification for patients.	Medication adherence not as outcome measure
5.	PubMed	McTigue KM, Conroy MB, Hess R, Bryce CL, Fiorillo AB, Fischer GS, Milas NC, Simkin-Silverman LR.	Using the internet to translate an evidence-based lifestyle intervention into practice.	Medication adherence not as outcome measure
6.	PubMed	Decker V, Spoelstra S, Miezo E, Bremer R, You M, Given C, Given B.	A pilot study of an automated voice response system and nursing intervention to monitor adherence to oral chemotherapy agents.	No internet intervention

7.	PubMed	Tamblyn R, Reidel K, Huang A, Taylor L, Winslade N, Bartlett G, Grad , Jacques A, Dawes M, Larochelle P, Pinsonneault A.	Increasing the detection and response to adherence problems with cardiovascular medication in primary care through computerized drug management systems: a randomized controlled trial.	Intervention not patient-centered
8.	PubMed	Yoo HJ, Park MS, Kim TN, Yang SJ, Cho GJ, Hwang TG, Baik SH, Choi DS, Park GH, Choi KM.	A Ubiquitous Chronic Disease Care system using cellular phones and the internet.	Medication adherence not as outcome measure
9.	PubMed	McCarrier KP, Ralston JD, Hirsch IB, Lewis G, Martin DP, Zimmerman FJ, Goldberg HI.	Web-based collaborative care for type 1 diabetes: a pilot randomized trial.	Medication adherence not as outcome measure
10.	PubMed	Cho JH, Lee HC, Lim DJ, Kwon HS, Yoon KH.	Mobile communication using a mobile phone with a glucometer for glucose control in Type 2 patients with diabetes: as effective as an Internet-based glucose monitoring system.	Full text could not be obtained
11.	PubMed	Lorig KR, Ritter PL, Dost A, Plant K, Laurent DD, McNeil I.	The Expert Patients Programme online, a 1-year study of an Internet-based self-management programme for people with long-term conditions.	Medication adherence not as outcome measure
12.	PubMed	Ralston JD, Hirsch IB, Hoath J, Mullen M, Cheadle A, Goldberg HI.	Web-based collaborative care for type 2 diabetes: a pilot randomized trial.	Medication adherence not as outcome measure
13.	PubMed	Cocosila M, Archer N, Haynes RB, Yuan Y.	Can wireless text messaging improve adherence to preventive activities? Results of a randomized controlled trial.	No chronic medication

14.	PubMed	Lawrence DB, Allison W, Chen JC, Demand M.	Improving medication adherence with a targeted, technology-driven disease management intervention.	No internet intervention
15.	PubMed	Faridi Z, Liberti L, Shuval K, Northrup V, Ali A, Katz DL.	Evaluating the impact of mobile telephone technology on type 2 diabetic patients' self-management: the NICHE pilot study.	Medication adherence not as outcome measure
16.	PubMed	Basheti IA, Armour CL, Bosnic-Anticevich SZ, Reddel HK.	Evaluation of a novel educational strategy, including inhaler-based reminder labels, to improve asthma inhaler technique.	Medication adherence not as outcome measure
17.	PubMed	Schulz PJ, Rubinell S, Hartung U.	An internet-based approach to enhance self-management of chronic low back pain in the Italian-speaking population of Switzerland: results from a pilot study.	Medication adherence not as outcome measure
18.	PubMed	Kim SI, Kim HS.	Effectiveness of mobile and internet intervention in patients with obese type 2 diabetes.	Medication adherence not as outcome measure
19.	PubMed	O'Shea SI, Arcasoy MO, Samsa G, Cummings SE, Thames EH, Surwit RS, Ortel TL.	Direct-to-patient expert system and home INR monitoring improves control of oral anticoagulation.	No internet intervention
20.	PubMed	Hee-Sung K.	Impact of Web-based nurse's education on glycosylated haemoglobin in type 2 Diabetic patients.	Medication adherence not as outcome measure
21.	PubMed	Kim HS, Jeong HS.	A nurse short message service by cellular phone in type-2 diabetic patients for six months.	Medication adherence not as outcome measure
22.	PubMed	Wangberg SC.	An Internet-based diabetes self-care intervention tailored to self-efficacy.	Medication adherence not as outcome measure

23.	PubMed	Wu AW, Snyder CF, Huang IC, Skolasky R, McGruder HF, Celano SA, Selnes OA, Andrade AS.	A randomized trial of the impact of a programmable medication reminder device on quality of life in patients with AIDS.	No internet intervention
24.	PubMed	Cho JH, Chang SA, Kwon HS, Choi YH, Ko SH, Moon SD, Yoo SJ, Song KH, Son HS, Kim HS, Lee WC, Cha BY, Son HY, Yoon KH.	Long-term effect of the Internet-based glucose monitoring system on HbA1c reduction and glucose stability: a 30-month follow-up study for diabetes management with a ubiquitous medical care system.	Medication adherence not as outcome measure
25.	PubMed	Gerber BS.	The chronic disease self-management program: extending reach through the internet.	Full text could not be obtained
26.	PubMed	Roumie CL, Elasy TA, Greevy R, Griffin MR, Liu X, Stone WJ, Wallston KA, Dittus RS, Alvarez V, Cobb J, Speroff T.	Improving blood pressure control through provider education, provider alerts, and patient education: a cluster randomized trial.	No patient centered intervention
27.	PubMed	Chatkin JM, Blanco DC, Scaglia N, Wagner MB, Fritscher CC.	Impact of a low-cost and simple intervention in enhancing treatment adherence in a Brazilian asthma sample.	No Internet intervention
28.	PubMed	Puccio JA, Belzer M, Olson J, Martinez M, Salata C, Tucker D, Tanaka D.	The use of cell phone reminder calls for assisting HIV-infected adolescents and young adults to adhere to highly active antiretroviral therapy: a pilot study.	No Internet intervention

29.	PubMed	Hornick TR, Higgins PA, Stollings C, Wetzel L, Barzilai K, Wolpaw D.	Initial evaluation of a computer-based medication management tool in a geriatric clinic.	Medication adherence not as outcome measure
30.	PubMed	Feldstein A, Elmer PJ, Smith DH, Herson M, Orwoll E, Chen C, Aickin M, Swain MC.	Electronic medical record reminder improves osteoporosis management after a fracture: a randomized, controlled trial.	Medication adherence not as outcome measure
31.	PubMed	Fonseca JA, Costa-Pereira A, Delgado L, Fernandes L, Castel-Branco MG.	Asthma patients are willing to use mobile and web technologies to support self-management.	Medication adherence not as outcome measure
32.	PubMed	Bray P, Roupe M, Young S, Harrell J, Cummings DM, Whetstone LM.	Feasibility and effectiveness of system redesign for diabetes care management in rural areas: the eastern North Carolina experience.	No patient centered intervention
33.	PubMed	Pines A.	Compliance with hormone therapy after Women's Health Initiative: who is to blame?	No intervention study
34.	PubMed	Ryan D, Cobern W, Wheeler J, Price D, Tarassenko L.	Mobile phone technology in the management of asthma.	No Internet intervention
35.	PubMed	Bush N, Donaldson G, Moinpour C, Haberman M, Milliken D, Markle V, Lauson J.	Development, feasibility and compliance of a web-based system for very frequent QOL and symptom home self-assessment after hematopoietic stem cell transplantation.	Medication adherence not as outcome measure
36.	PubMed	Hagström B, Mattsson B, Rost IM, Gunnarsson RK.	What happened to the prescriptions? A single, short, standardized telephone call may increase compliance.	No Internet intervention

37.	PubMed	Kwon HS, Cho JH, Kim HS, Song BR, Ko SH, Lee JM, Kim SR, Chang SA, Kim HS, Cha BY, Lee KW, Son HY, Lee JH, Lee WC, Yoon KH.	Establishment of blood glucose monitoring system using the internet.	No intervention study
38.	PubMed	Fairley CK, Levy R, Rayner CR, Allardice K, Costello K, Thomas C, McArthur C, Kong D, Mijch A, Melbourne Adherence Group; Melbourne Adherence Group.	Randomized trial of an adherence programme for clients with HIV.	No Internet intervention
39.	PubMed	Larsen DL, Cannon W, Towner S.	Longitudinal assessment of a diabetes care management system in an integrated health network.	Medication adherence not as outcome measure
40.	PubMed	Cramer J, Rosenheck R, Kirk G, Krol W, Krystal J; VA Naltrexone Study Group 425.	Medication compliance feedback and monitoring in a clinical trial: predictors and outcomes.	No patient centered intervention
41.	PubMed	Safren SA, Hendriksen ES, Desousa N, Boswell SL, Mayer KH.	Use of an on-line pager system to increase adherence to antiretroviral medications.	No Internet intervention
42.	PubMed	Akron General Medical Center, Akron, Ohio 44333, USA. shughes@agmc.org	The use of non face-to-face communication to enhance preventive strategies.	No intervention study

43.	PubMed	McAlindon T, Formica M, Kabbara K, LaValley M, Lehmer M.	Conducting clinical trials over the internet: feasibility study.	No chronic medication
44.	PubMed	Hart T, Hawkey K, Whyte J.	Use of a portable voice organizer to remember therapy goals in traumatic brain injury rehabilitation: a within-subjects trial.	Medication adherence not as outcome measure
45.	PubMed	Stuart GW, Laraia MT, Ornstein SM, Nietert PJ.	An interactive voice response system to enhance antidepressant medication compliance.	No Internet intervention
46.	PubMed	Burkhart PV, Dunbar-Jacob JM, Fireman P, Rohay J.	Children's adherence to recommended asthma self-management.	No Internet intervention
47.	PubMed	Finkelstein J, O'Connor G, Friedmann RH.	Development and implementation of the home asthma telemonitoring (HAT) system to facilitate asthma self-care.	Medication adherence not as outcome measure
48.	PubMed	Andrade A.	HIV adherence strategies take a high-tech route.	Full text could not be obtained
49.	PubMed	Frances CD, Alperin P, Adler JS, Grady D.	Does a fixed physician reminder system improve the care of patients with coronary artery disease? A randomized controlled trial.	No Internet intervention
50.	PubMed	Bennett SJ, Hays LM, Embree JL, Arnould M.	Heart Messages: a tailored message intervention for improving heart failure outcomes.	No intervention study
51.	PubMed	Johnson BF, Hamilton G, Fink J, Lucey G, Bennet N, Lew R.	A design for testing interventions to improve adherence within a hypertension clinical trial.	No Internet intervention
52.	PubMed	Curtin K, Hayes BD, Holland CL, Katz LA.	Computer-generated intervention for asthma population care management.	Medication adherence not as outcome measure

53.	PubMed	Cramer JA, Rosenheck R.	Enhancing medication compliance for people with serious mental illness.	No Internet intervention
54.	PubMed	Legorreta AP, Hasan MM, Peters AL, Pelletier KR, Leung KM.	An intervention for enhancing compliance with screening recommendations for diabetic retinopathy. A bicoastal experience.	Medication adherence not as outcome measure
55.	PubMed	Milch RA, Ziv L, Evans V, Hillebrand M.	The effect of an alphanumeric paging system on patient compliance with medicinal regimens.	No Internet intervention
56.	PubMed	Casebeer L, Roesener GH.	Patient informatics: using a computerized system to monitor patient compliance in the treatment of hypertension.	Full text could not be obtained
57.	PubMed	Schectman G, Hiatt J, Hartz A.	Telephone contacts do not improve adherence to niacin or bile acid sequestrant therapy.	No Internet intervention
58.	PubMed	Skaer TL, Sclar DA, Markowski DJ, Won JK.	Effect of value-added utilities on prescription refill compliance and health care expenditures for hypertension.	No Internet intervention
59.	PubMed	Turner BJ, Day SC, Borenstein B.	A controlled trial to improve delivery of preventive care: physician or patient reminders?	No Internet intervention
60.	PubMed	Simkins CV, Wenzloff NJ.	Evaluation of a computerized reminder system in the enhancement of patient medication refill compliance.	No Internet intervention
61.	PubMed	Ascione FJ, Brown GH, Kirking DM.	Evaluation of a medication refill reminder system for a community pharmacy.	No Internet intervention
62.	PubMed	Heard C, Blackburn JL, Thompson MS, Wallace SM.	Evaluation of a computer-assisted medication refill reminder system for improving patient compliance.	No Internet intervention
63.	EMBASE	Viera AJ, Jamieson B	How effective are hypertension self-care interventions	No Internet intervention

64.	EMBASE	Shanovich KK, Sorkness CA, wise M, Pulvermacher AD, Bhattacharya A, Gustafson DH.	Internet telehealth for pediatric nurse case management improves asthma control.	No patient centered intervention
65	EMBASE	Benhamou PY, Melki V, Boizel R, Perreal F, Quesada JL, Bessieres-Lacombe S *et al*.	One-year efficacy and safety of Web-based follow-up using cellular phone in type 1 diabetic patients under insulin pump therapy: the PumpNet study	No Internet intervention
66.	EMBASE	Bond GE, Burr R, Wolf FM, Price M, McCurry SM, Teri L.	The effects of a web-based intervention on the physical outcomes associated with diabetes among adults age 60 and older: A randomized trial.	Medication adherence not as outcome measure
67.	EMBASE	McMahon GT, Gomes HE, Hohne SH, Hu TMJ, Levine BA, Conlin PR.	Web-based care management in patients with poorly controlled diabetes.	Medication adherence not as outcome measure
68.	CINAHL	Phillips A.	Web based care management improved glucose control in patients with poorly controlled diabetes.	Medication adherence not as outcome measure
69.	CINAHL	Pontin D.	An interactive monitoring device reduced asthma symptoms and functional limitations in inner city children with asthma	Medication adherence not as outcome measure
