# Supplementary material for: Effects of eHealth Interventions on Medication Adherence: A Systematic Review of the Literature
Source: J Med Internet Res. 2011 Dec 5;13(4):e103. doi: 10.2196/jmir.1738 (PMC3278089; doi:10.2196/jmir.1738)
Supplement: Supplementary file 2 [file jmir_v13i4e103_app2.pdf]

Multimedia Appendix 2

Study & Method	Type of disease	Type of intervention	Delivering of tailored message	Participants, Sex (%); mean age Groups	Name of instrument adherence, moment of measuring adherence	Conclusions
Artinian [27]<br>RCT	Patients with symptomatic left ventricular dysfunction	A web-based monitoring system	- Tailored content<br>- Nature of expert/therapist contact	N=18<br>Mean age: 68<br><br>17 males<br><br>Intervention group n=9<br>Control group n=9	- Pill counts<br>-Baseline and 3 months	Medication compliance rate was 94% for the monitor group as measured by the monitor system
Jan [28]<br>RCT	Paediatric asthma patients	Blue Angel for Asthma Kids variability	- Tailored content<br><br>- Nature of expert/therapist contact	N = 164<br><br>Intervention group n= 88<br><br>Mean age 10.9<br>35 males<br>Control group n=76<br>Mean age 9.9<br>28 males	- Self-reported use and test score for dry powder inhaler (DPI) or metered dose inhaler (MDI) with the spacer technique<br>- Baseline and 12 weeks	The Blue Angel for Asthma Kids, has the potential for improving asthma outcome compared with conventional treatment over a period of 12 weeks
Chan [29]<br>RCT	Paediatric asthma patients	A customized educational and monitoring Web site	- Tailored content<br><br>- Nature of expert/therapist contact	N=120<br><br>Intervention group n=60<br><br>Mean age 10.2<br>37 males<br>Control group n= 60<br>Mean age 9.0<br>38 males	Computerized prescription refill record<br>- Baseline, 26 and 52 weeks	No difference in adherence between both groups

Chan [30]<br>RCT	Paediatric asthma patients	A customized educational and monitoring Web site	- Tailored content<br><br>- Nature of expert/therapist contact	N=10<br><br>Mean age: 7<br><br>5 males<br>Intervention group n=5<br>Mean age 6.6<br><br>1 male<br>Control group N=5<br>Mean age 8.7<br>4 males	- Self reported asthma diary and computerized prescription refill record<br><br>- 90 days and 180 days	After the intervention, the use of β-agonist decreased, which is an indication of better adherence
Joseph [31]<br><br>RCT	Patients with asthma	Web-based asthma management program	- Tailored content<br><br>- User control	N=314<br><br>Mean age 15.3<br>36.6% male<br><br>Intervention group: n= 162<br>Control group n= 152	- Self-reported<br><br>- Baseline and 12 months	Positive changes in controller medication adherence
Ross [32]<br><br>RCT	Patients with Heart Failure	The SPPARO (System Providing Access to Records Online)	- Tailored content<br><br>- Nature of expert/therapist contact	N= 104<br><br>Intervention group n= 54<br><br>Mean age 57<br><br>80% male<br>Control group n=50<br>Mean age 55<br>74% male	- Self-reported<br><br>- Baseline, 6 months, 12 months	Providing patients access to an online medical record improved adherence
Cherry [33]<br><br>Prospective design	Patients with diabetes	Telemedicine diabetes disease management program.	- Tailored content<br><br>- Nature of expert/therapist contact	Intervention group n=169<br><br>Mean age 53<br><br>39 males<br><br>Historical group (usual care)	- Self-reported<br><br>- Baseline, 12 months	Outcomes offer encouraging evidence that telemedicine technology coupled with daily remote monitoring may improve appropriate utilization of healthcare services

Guendelman [34]<br>RCT	Persistent asthma.	Health Buddy, an interactive device connected to a home telephone	- Tailored content<br><br>- Nature of expert/therapist contact	N=134<br><br>Intervention group mean age 12.0<br>40 male<br>Control group Mean age 12.2<br>37 male	- Self-reported<br>- Baseline, 6 and 12 weeks	Patients were more likely to take their asthma medication without additional reminders
DeVito Dabbs [35]<br>RCT	Patients who received a lung transplantation	Pocket Personal Assistant for Tracking Health	- Tailored content<br><br>- Nature of expert/therapist contact	N = 30<br><br>Intervention group n=15<br><br>Mean age 55<br>60% male<br>Control group n=15<br>Mean age 57<br>60% male	- Self reported<br>- Baseline, 2 months	Patients who received the PATH were more likely to show high adherence to the medical regimen
Van der Meer [36]<br>RCT	Patients with asthma	Internet-based self management programme	- Customized Health program<br>- User control	N = 200<br><br>Intervention Group n= 101<br><br>Mean age 36<br>29% male<br>Control Group n=99<br>Mean age 37<br>29% male	Adherence: self-reported. Baseline, 3 months and 6 months	In the first 3 months, many patients had uncontrolled asthma and were advised to increase their inhaled corticosteroid doses. The improvement in asthma control seen after 3 months allowed a decrease in inhaled corticosteroid medication over the next 9 months without loss of asthma control. This pattern suggests tailoring medication to patients' needs rather than increasing medication for the whole study sample
Van der Meer [37]<br>RCT	Patients with mild to moderate persistent asthma	Internet-based self management programme	- Customized Health program<br>- User control	N= 200<br><br>Intervention n=111<br><br><br>Mean age 36<br>28 males<br>Group 1 (well controlled)	- Self-reported<br>- Baseline and after 3 months and 1 year	Weekly self-monitoring and subsequent treatment adjustment leads to improved asthma control in patients with partly and uncontrolled asthma at baseline and

				n=37<br>Group 2 (partly controlled)<br>n=38<br>Group 3 (uncontrolled)<br>n=36<br>Control group<br>n = 89<br>Mean age 36.6<br>28 male<br>Group 1 (well controlled)<br>n=38<br>Group 2 (partly controlled)<br>n=33<br>Group 3 (uncontrolled)<br>n=28		tailors asthma medication to individual patients' needs
Dilorio [38]<br><br>Survey	Patients with Epilepsy	WebEase	- Customized Health Program<br><br>- User control	N = 35<br><br>Mean age 37.5<br>40% male	- Self-reported<br><br>- Baseline and 6 weeks	Participants showed some improvement in adherence following the program
Dew [39]<br><br>Prospective design	Heart recipients and their family caregivers	Website including skills workshops, discussion group, ask an expert, question and answer, health tips, recourses and references	- Customized Health Program<br>- Nature of expert/therapist contact.	N=64<br><br>Intervention group n= 24<br><br>Mean age 45.8<br>18 males<br>Control group n= 40<br>Mean age = 57.5<br>30 males	- Self-reported<br>- Baseline, 4 months	The intervention appeared to be most weakly associated with medical compliance change
